# Power MOSFET Linearizer of a High-Voltage Power Amplifier for High-Frequency Pulse-Echo Instrumentation

**DOI:** 10.3390/s17040764

**Published:** 2017-04-04

**Authors:** Hojong Choi, Park Chul Woo, Jung-Yeol Yeom, Changhan Yoon

**Affiliations:** 1 Department of Medical IT Convergence Engineering, Kumoh National Institute of Technology, Gumi 39253, Korea; hojongch@kumoh.ac.kr (H.C.); pcw3108@kumoh.ac.kr (P.C.W.); 2 School of Biomedical Engineering, Korea University, Seoul 02841, Korea; 3 Department of Biomedical Engineering, Inje University, Gimhae 50834, Korea

**Keywords:** high-voltage power amplifier, ultrasonic transducer, power MOSFET linearizer

## Abstract

A power MOSFET linearizer is proposed for a high-voltage power amplifier (HVPA) used in high-frequency pulse-echo instrumentation. The power MOSFET linearizer is composed of a DC bias-controlled series power MOSFET shunt with parallel inductors and capacitors. The proposed scheme is designed to improve the gain deviation characteristics of the HVPA at higher input powers. By controlling the MOSFET bias voltage in the linearizer, the gain reduction into the HVPA was compensated, thereby reducing the echo harmonic distortion components generated by the ultrasonic transducers. In order to verify the performance improvement of the HVPA implementing the power MOSFET linearizer, we measured and found that the gain deviation of the power MOSFET linearizer integrated with HVPA under 10 V DC bias voltage was reduced (−1.8 and −0.96 dB, respectively) compared to that of the HVPA without the power MOSFET linearizer (−2.95 and −3.0 dB, respectively) when 70 and 80 MHz, three-cycle, and 26 dB_m_ input pulse waveforms are applied, respectively. The input 1-dB compression point (an index of linearity) of the HVPA with power MOSFET linearizer (24.17 and 26.19 dB_m_ at 70 and 80 MHz, respectively) at 10 V DC bias voltage was increased compared to that of HVPA without the power MOSFET linearizer (22.03 and 22.13 dB_m_ at 70 and 80 MHz, respectively). To further verify the reduction of the echo harmonic distortion components generated by the ultrasonic transducers, the pulse-echo responses in the pulse-echo instrumentation were compared when using HVPA with and without the power MOSFET linearizer. When three-cycle 26 dB_m_ input power was applied, the second, third, fourth, and fifth harmonic distortion components of a 75 MHz transducer driven by the HVPA with power MOSFET linearizer (−48.34, −44.21, −48.34, and −46.56 dB, respectively) were lower than that of the HVPA without the power MOSFET linearizer (−45.61, −41.57, −45.01, and −45.51 dB, respectively). When five-cycle 20 dB_m_ input power was applied, the second, third, fourth, and fifth harmonic distortions of the HVPA with the power MOSFET linearizer (−41.54, −41.80, −48.86, and −46.27 dB, respectively) were also lower than that of the HVPA without the power MOSFET linearizer (−25.85, −43.56, −49.04, and −49.24 dB, respectively). Therefore, we conclude that the power MOSFET linearizer could reduce gain deviation of the HVPA, thus reducing the echo signal harmonic distortions generated by the high-frequency ultrasonic transducers in pulse-echo instrumentation.

## 1. Introduction

A pulse-echo instrumentation typically consists of ultrasonic transmitters, receivers, and transducers [1,2,3]. In the ultrasonic transmitters, the high voltage power amplifier (HVPA) is one of the main electronic components since the HVPA triggers the ultrasonic transducers through the expander devices, which consist of cross-coupled diodes [2,4]. Therefore, the performance improvements of the HVPA, such as gain, bandwidth, and linearity have been one of the critical technical issues. Especially, linearity is an important parameter for HVPA used in the ultrasound transmitters because the non-linear HVPA leads to high harmonic distortions, thus deteriorating the echo signal quality of the ultrasonic transducers [5].

**Figure 1:**
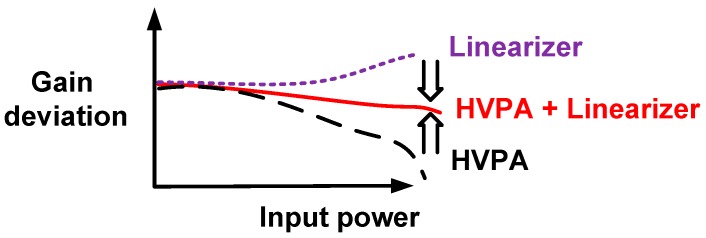
Gain deviation of HVPA with and without the linearizer as a function of input power.

For high-frequency ultrasonic transducers compared to low-frequency ultrasonic transducers, the size of the piezoelectric materials (the main material of the ultrasonic transducers) is small, thus reducing the capacitance values of the piezoelectric materials as the operating frequency of the ultrasonic transducers are increased [6,7]. Therefore, the parasitic capacitances of the coaxial cables can affect the performances of the high-frequency ultrasonic transducers. In addition, the maximum excitation voltages into the high-frequency ultrasonic transducers are, accordingly, limited due to the size reduction of the piezoelectric materials [8]. These external interventions affect the performances of high-frequency pulse-echo instrumentation. Therefore, linearizing techniques, such as compensating for several non-linear active and passive electronic components in the HVPA, could help improve HVPA linearity, thus improving the performance of high-frequency pulse-echo instrumentations. The basic operating principle of the linearizer and HVPA is described in Figure 1. As depicted in the figure, the gain of the HVPA typically decreases as the input power increases due to the sensitive parasitic impedances [9,10,11,12].

Linearizer techniques have been widely researched in RF communication applications [11]. However, the linearizer techniques have only been recently introduced in the ultrasound research [13,14]. The systematic approaches use the analog-to-digital converter, digital-to-analog converter, and memory spaces, which lead to increased cost, larger size, and higher complexity [13]. Therefore, it is not preferable for implementation into ultrasound array transducer-based systems. The pre-linearizer technique provides low cost, small size, and low complexity compared to the systematic approaches [11]. However, the reported pre-linearizer requires complex mathematical model analysis to compensate the non-linearity in the HVPA and it is not suitable for the ultrasound HVPA utilizing input and output DC coupling capacitors [14]. Therefore, we develop a simple linearizer circuit to be used for HVPA ultrasound applications. As shown in Figure 1, the linearizers need to be designed to have opposite characteristics with the HVPA. Therefore, the linearizer gain deviation should be positively increased to reduce the gain deviation of the HVPA.

The HVPA in ultrasonic transmitters usually utilizes large-value coupling capacitors in order to block DC voltages which in turn deteriorate the echo signal quality of the performances of high-frequency pulse-echo instrumentations like high harmonic distortion components of the echo signals because the coupling capacitors reduce the gain of the HVPA at high input powers [2,15,16]. In a typical HVPA topology, large-value coupling capacitors in the input and output ports are required to block DC voltages as they can affect the echo signal quality of the ultrasonic transducers. Unfortunately, these coupling capacitors are inherently non-linear passive components such that it also generates unwanted harmonic distortions. Therefore, the harmonic distortions generated from HVPA directly affect the performance of ultrasonic transducers. In order to reduce the harmonic distortion components of the echo signal generated by the HVPA, we propose employing simple linearizer techniques utilizing variable power MOSFET devices with shunt inductors and capacitors. The detailed architecture, operating mechanism, and implementation of the linearizer with an HVPA are described in Section 2. The capability to reduce the harmonic distortions of the echo signals of the ultrasonic transducers are verified with the developed linearizer with an HVPA in Section 3. The conclusion of the paper is given in Section 4.

## 2. Materials and Methods

**Figure 2:**
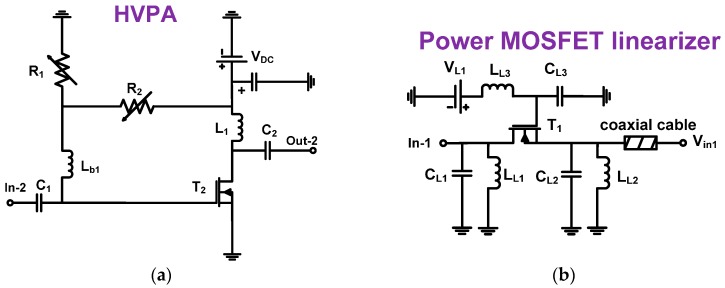
Schematic diagrams of (**a**) HVPA and (**b**) power MOSFET linearizer.

Figure 2a,b show schematic diagrams of the HVPA and the power MOSFET linearizer. In the HVPA architecture (Figure 2a), the HVPA is a Class A-type power amplifier used in the ultrasound applications. The large value 100 pF DC coupling capacitors (C_1_ and C_2_) are used to block DC voltage. The two variable 2 kΩ resistors (R_1_ and R_2_) are used to set the bias voltage of the high-voltage transistor (T_2_). The 3.3 μH RF choke inductor (L_1_) is used to maximize the voltage amplification of the HVPA. The 2 μH inductor (L_b1_) is used to prevent bias voltage reduction. A 28 V DC voltage (V_DC_) is supplied from a power supply. In a typical HVPA topology, a large value RF choke inductor is required to reduce the DC voltage drop from the power supply (V_DC_), thus allowing the output voltage of the HVPA to be amplified without some suppression because the maximum output voltage is limited by the DC power supply [12]. If a large value resistor is used instead of the large value RF choke inductor, the power supply DC voltage drop could affect the maximum output voltage amplification. Bias inductors with large values are also needed to set the DC bias voltage after the resistor divider circuit (R_1_ and R_2_). Therefore, the maximum DC gate bias voltage is generated by the resistor divider circuits. If we use a resistor instead of the inductor, there would be an additional DC voltage drop and that could limit the output voltage amplification [12].

As shown in Figure 2b, we designed a power MOSFET linearizer which consists of shunt capacitors and inductors with power MOSFET devices working with variable DC bias voltages. The 3.3 μH inductor value (L_L3_) is used to minimize the voltage drop from the power supply (V_L1_). The 100 pF capacitor (C_L3_) is used to apply stable DC bias voltage to the power MOSFET device (IRF620, Vishay Siliconix, Malvern, PA, USA) because DC power noise signals could go to ground. Two capacitors (C_L1_ and C_L2_) are used to short possible unwanted AC noise from the power supply (V_L1_). The 50-Ω coaxial cable is used to minimize the signal fluctuation between the power MOSFET linearizer and the HVPA.

**Figure 3:**
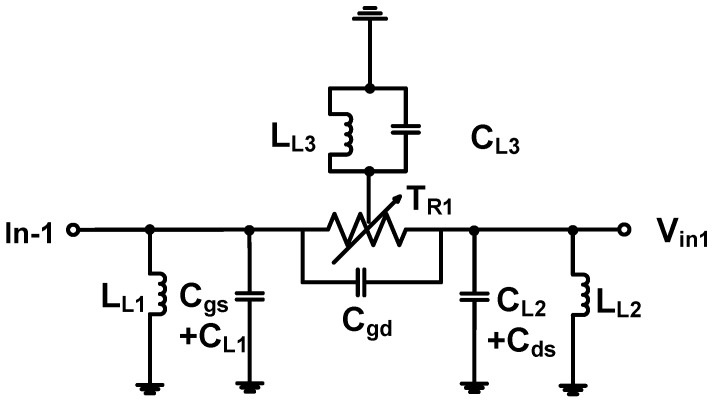
The equivalent circuit model of the power MOSFET linearizer.

Figure 3 shows the equivalent circuit model of the power MOSFET linearizer. As shown in Figure 3, depending on the gate bias voltage, the MOSFET in the linearizer works as a variable resistor (T_R1_) to provide variable performance when used in conjunction with two fixed 20 pF and 100 nH values of capacitors (C_L1_ and C_L2_) and inductors (L_L1_ and L_L2_). These capacitors are bypass capacitors to block possible noise at the input port generated by the function generator and the power supply [12,17]. Low frequency ultrasound applications may not require bypass capacitors, however, high-frequency ultrasound applications are very sensitive to the performance of the HVPA due to the limited maximum output power of the ultrasonic transducers [8]. However, as large capacitance can affect the performance of the circuit, and the MOSFET (T_1_) inherently has large parasitic capacitance, smaller value bypass capacitor are preferred here. Therefore, the 20 pF capacitor value is selected to be much less than the parasitic capacitances of the MOSFET (input and output capacitances = 230 and 70 pF). The total output of the power MOSFET linearizer can be expressed by:

(1) $$\frac{V_{in1}}{V_{in-1}}\approx \frac{\left[\frac{1}{\frac{1}{jwL_{L2}}+jwC_{ds}}\right]}{\left[\frac{1}{jwC_{gd}+\frac{1}{T_{R1}}}\right]+\left[\frac{1}{\frac{1}{jwL_{L2}}+jwC_{ds}}\right]}$$

where *w* is the operating frequency.

As shown in Equation (1), the performance of the power MOSFET linearizer is determined by the variable resistor of the MOSFET (T_R1_) and inductor (L_L2_) because the MOSFET device has larger pre-determined parasitic capacitances (C_gd_ and C_ds_) compared to the two capacitors (C_L1_ and C_L2_). Therefore, the performance of the power MOSFET linearizer with different inductor values (L_L2_) were measured in order to find the appropriate operating frequencies of the power MOSFET linearizer because the power MOSFET linearizer could reduce the gain of the HVPA at certain operating frequencies. The 100, 33, 22, and 12 nH inductors were selected to have minimum loss of the MOSFET linearizer at 50, 70, 80, and 90 MHz operating frequencies. We selected a 33 nH inductance value to minimize the signal loss (lower than −3.8 dB) measured at 70 and 80 MHz. With the selected inductor values, the performances of the MOSFET linearizer could essentially be controlled depending on the MOSFET resistance (T_R1_). In Section 3, the power MOSFET linearizer is characterized because the variable resistances of the MOSFET can be varied under different DC values (V_L1_) as shown in Figure 2b.

## 3. Results and Discussion

### 3.1. Linearizer Performance Measurement

**Figure 4:**
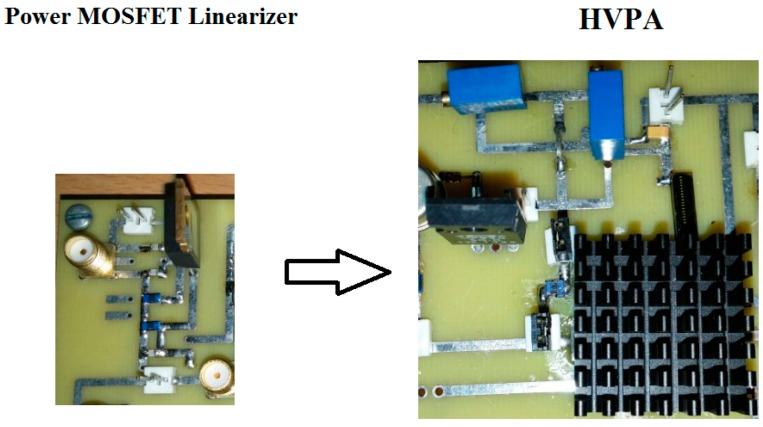
The implemented power MOSFET linearizer and HVPA on a printed circuit board.

**Figure 5:**
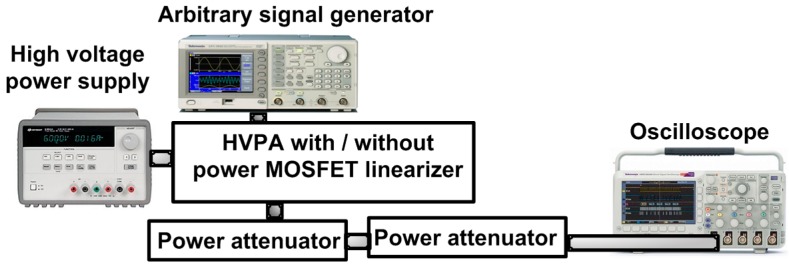
The measurement setup for HVPA with and without the power MOSFET linearizer.

The HVPA and power MOSFET linearizer were implemented on a printed circuit board with two layers. In the HVPA, the heat-sink was attached on top of the high-voltage transistors as shown in Figure 4. Our target ultrasonic transducer frequency for the pulse-echo instrumentations is over 50 MHz, as it is known that in studies with the eye, intravascular, acoustic stimulation, and cavitation, high-frequency (over 50 MHz) ultrasonic applications have reported better spatial resolutions compared to low-frequency (less than 15 MHz) ultrasonic applications [18,19,20,21]. Figure 5 shows the experimental setup to measure the gain deviation at 1-dB compression point of the HVPA with and without the power MOSFET linearizer in order to acquire the proper working bias voltages of the power MOSFET linearizer. The gate-threshold voltage of the MOSFET in the power MOSFET linearizer is typically between 2 V and 4 V, depending on the drain-source voltage such that the power MOSFET linearizer performs as a variable resistor over the gate-threshold voltages. The three-cycle 70 or 80 MHz input pulses generated from an arbitrary signal generator (AFG3252, Tektronix Inc., Beaverton, OR, USA) were applied to the HVPA without and with the power MOSFET linearizer under preset DC biasing with a high-voltage power supply (E3631A, Agilent Technologies, Santa Clara, CA, USA). The output was then fed through two 20 dB power attenuators (BW-S20W20+, Mini-circuits, Brooklyn, NY, USA) and acquired with an oscilloscope (MSOX4154A, Keysight Technology, Santa Clara, CA, USA) to show the gain deviations of the HVPA with and without the power MOSFET linearizer.

**Figure 6:**
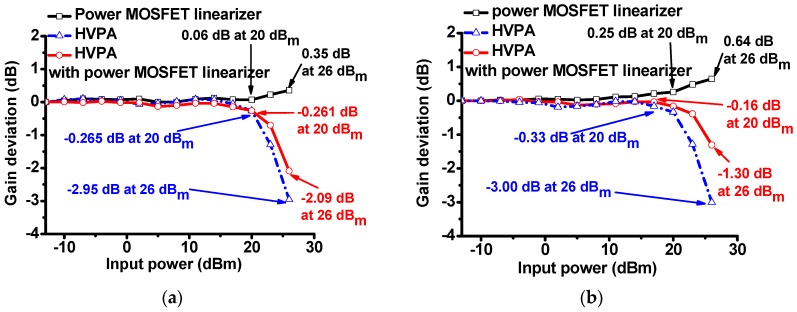
The gain deviation graphs of the power MOSFET linearizer, HVPA, and HVPA with power MOSFET linearizer when (**a**) 70 and (**b**) 80 MHz input power were applied.

In order to assess the power MOSFET linearizer characteristics, we measured the voltage gain deviation graphs of the HVPA with and without the power MOSFET linearizer at 70 and 80 MHz under 5 V DC bias. The gain deviation graphs of (1) the power MOSFET linearizer; (2) the HVPA only, and (3) the HVPA with the power MOSFET linearizer at 70 MHz input power are plotted in Figure 6a. The measured gain deviations of the power MOSFET linearizer and HVPA at 20 and 26 dB_m_ input power were 0.06 and 0.35 dB, and −0.265 and −2.95 dB, respectively. The gain deviations of the HVPA with the power MOSFET linearizer reduced to −0.261 and −2.09 dB, respectively. The gain deviation graph of the MOSFET linearizer, HVPA, and HVPA with the power MOSFET linearizer at 70 MHz input power is plotted in Figure 6b. The measured gain deviations of the linearizer at 20 and 26 dB_m_ input power were 0.25 and 0.64 dB, respectively, and those of the HVPA at 20 dB_m_ and 26 dB_m_ input power were −0.33 and −3.00 dB, respectively. The gain deviations of the HVPA with a power MOSFET linearizer improved to −0.16 and −1.30 dB, respectively. From these results, we can conclude that the gain deviation of the HVPA with the MOSFET linearizer was suppressed as the input power increased. The difference in results when 70 and 80 MHz input power were applied to the power MOSFET linearizer is due to the non-linear frequency-dependent power MOSFET device parameters (total parasitic capacitances and inductance in the equivalent circuit model of the power MOSFET devices) shown in Figure 3. As derived in Equation (1), the frequency-dependent gain characteristic of the power MOSFET linearizer is dependent on the inductance (L_L2_) and two parasitic capacitances (C_ds_ and C_gd_).

**Figure 7:**
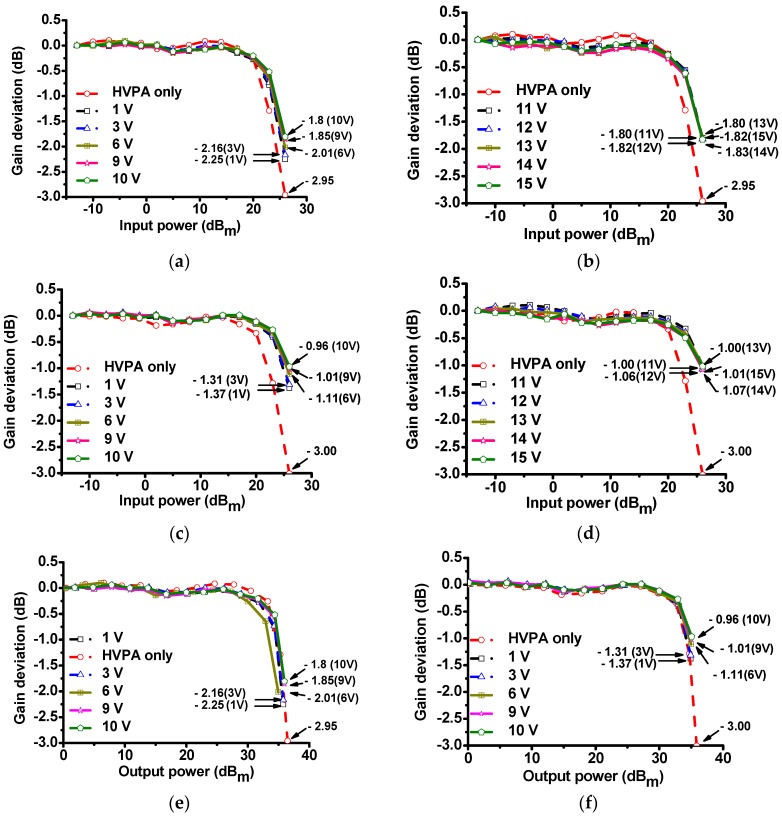
Normalized gain deviation of the HVPA with and without a power MOSFET linearizer vs. input power at (**a**) 70 MHz under DC bias voltages (1, 3, 6, 9, and 10 V DC, respectively), (**b**) under DC bias voltages (11, 12, 13, 14, and 15 V DC, respectively), at (**c**) 80 MHz under DC bias voltages (1, 3, 6, 9, and 10 V DC, respectively), at (**d**) 80 MHz under DC bias voltages (11, 12, 13, 14, and 15 V DC, respectively). Normalized gain deviation of the HVPA with and without a power MOSFET linearizer vs. output power at (**e**) 70 MHz under DC bias voltages (1, 3, 6, 9, and 10 V DC, respectively), and (**f**) at 80 MHz under DC bias voltages (1, 3, 6, 9, and 10 V DC, respectively).

Figure 7 shows the gain deviation graphs of the HVPA with and without the MOSFET linearizer under several DC bias voltages at 70 and 80 MHz, respectively. In Figure 7a, when 70 MHz and 26 dB_m_ input powers were applied to the power MOSFET linearizer, the gain deviation values under 6, 9, and 10 V DC bias conditions (−2.01, −1.85, and −1.80 dB, respectively) were reduced compared to 1 and 3 V DC bias conditions (−2.25 and −2.16 dB, respectively). In Figure 7b, when 70 MHz and 26 dB_m_ input powers were applied to the power MOSFET linearizer, the gain deviation under 11, 12, 13, 14, and 15 V DC bias conditions (−1.80, −1.82, −1.80, −1.83, and −1.82 dB, respectively) were still lower compared to that of the HVPA (−2.95 dB). In Figure 7a,b, when 70 MHz and 26 dB_m_ input power were applied to the HVPA with and without the power MOSFET linearizer, the gain deviation values of the HVPA with the power MOSFET linearizer were higher than that of the HVPA without the MOSFET linearizer (−2.95 dB). In Figure 7b, when 80 MHz and 26 dB_m_ input powers were applied to the power MOSFET linearizer, the gain deviation values under 6, 9, and 10 V DC bias conditions (−1.11, −1.01, and −0.96 dB, respectively) were reduced when compared to 1 and 3 V DC bias conditions (−1.37 and −1.31 dB, respectively). In Figure 7d, when 80 MHz and 26 dB_m_ input powers were applied to the power MOSFET linearizer, the gain deviation under 11, 12, 13, 14, and 15 V DC bias conditions were −1.00, −1.06, −1.00, −1.07, and −1.01 dB, respectively. In Figure 7c,d, when 80 MHz and 26 dB_m_ input power were applied to the HVPA with and without the power MOSFET linearizer, the gain deviation values of the HVPA with the power MOSFET linearizer were higher than that of the HVPA without the power MOSFET linearizer (−3.00 dB). Figure 7e,f show the normalized gain deviation of the HVPA with and without the MOSFET linearizer vs. the output power under 1, 3, 6, 9, and 10 V DC bias conditions, when 70 and 80 MHz input were applied to the power MOSFET linearizer respectively. As shown in Figure 7, the gain deviation performances of the HVPA improved with the help of the power MOSFET linearizer over high input power ranges. These experimental results illustrate that the gain deviation of the HVPA with the power MOSFET linearizer can be altered by controlling the DC bias feed voltages of the MOSFET. This fact is also meaningful because high-frequency ultrasonic transducers requires a relatively higher input power from the HVPA to achieve reasonable echo sensitivity compared to low-frequency ultrasonic transducers [1,4,8].

**Figure 8:**
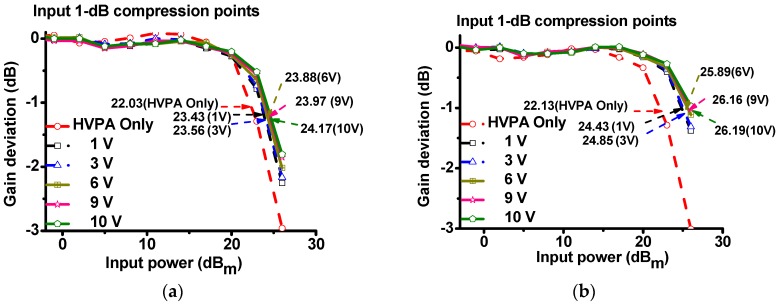
Measured 1-dB compression point graphs of the HVPA with and without power MOSFET linearizer at (**a**) 70 MHz and (**b**) 80 MHz under DC bias voltages (1, 3, 6, 9, and 10 V DC, respectively).

The input 1-dB compression point (IP_1dB_) provides an index of the linearity of the power amplifier components [22]. In fact, HVPA with higher IP_1dB_ has lower gain suppression than that with lower IP_1dB_. Figure 8 summarizes the measured IP_1dB_ of the HVPA with and without the power MOSFET linearizer at 70 and 80 MHz, respectively. As shown in Figure 8a, when 70 MHz, 26 dB_m_ and 3-cycle pulsed powers were sent to the HVPA with and without the power MOSFET linearizer, the measured IP_1dB_ of the HVPA without the power MOSFET linearizer (22.03 dB_m_) was lower than that with the power MOSFET linearizer under 10 V DC bias voltage (24.17 dB_m_). In Figure 8b, when the 80 MHz, 26 dB_m_, and three-cycle pulsed powers were sent to the HVPA with and without the power MOSFET linearizer, the measured IP_1dB_ of the HVPA without the power MOSFET linearizer (22.13 dB_m_) was lower than that with power MOSFET linearizer under 10 V DC bias voltage (26.19 dB_m_). Therefore, we confirm that the power MOSFET linearizer increased the IP_1dB_ of the HVPA.

Table 1 summarizes the performances of the HVPA with and without the linearizer at 70 and 80 MHz under different DC bias voltages.

### 3.2. High-Frequency Pulse-Echo Instrumentation

**Figure 9:**
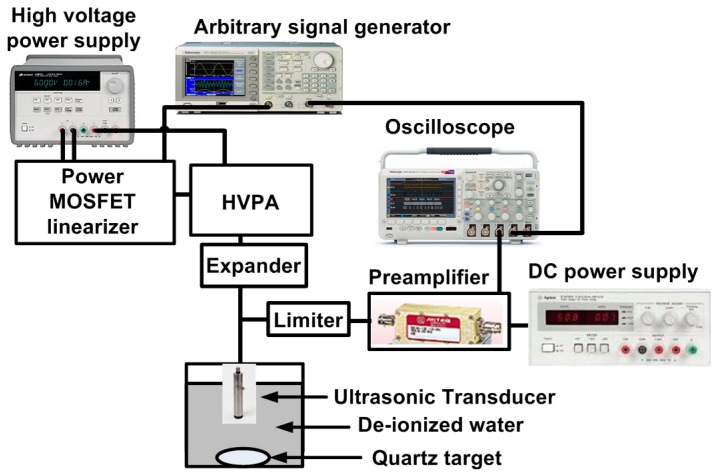
The measurement setup for the high frequency pulse-echo instrumentation.

Figure 9 shows the measurement setup for high-frequency pulse-echo instrumentation using the HVPA with the power MOSFET linearizer. The DC voltages generated by a high-voltage power supply (E3631A, Agilent Technologies, San Jose, CA, USA) were applied to the power MOSFET linearizer and HVPA and the pulse signals were generated by an arbitrary signal generator (AFG3252C, Tektronix Inc., Beaverton, OR, USA) were fed to the HVPA. The amplified high-voltage signals from the HVPA are transmitted through an expander into the ultrasonic transducer. The acoustic echo signals reflected from a quartz target were converted to the electrical echo signals by the ultrasonic transducers. Meanwhile, the discharged high-voltage signals sent from HVPA are blocked by the limiter before the electrical echo signals pass through the limiter. The echo signals were amplified by a preamplifier (AU-1114, L3 Technologies Inc., New York, NY, USA) with a DC power supply (E3630A, Agilent Technologies) and displayed the echo signals and their spectra on the oscilloscope (MSOX4154A, Keysight Technology, Santa Clara, CA, USA).

**Figure 10:**
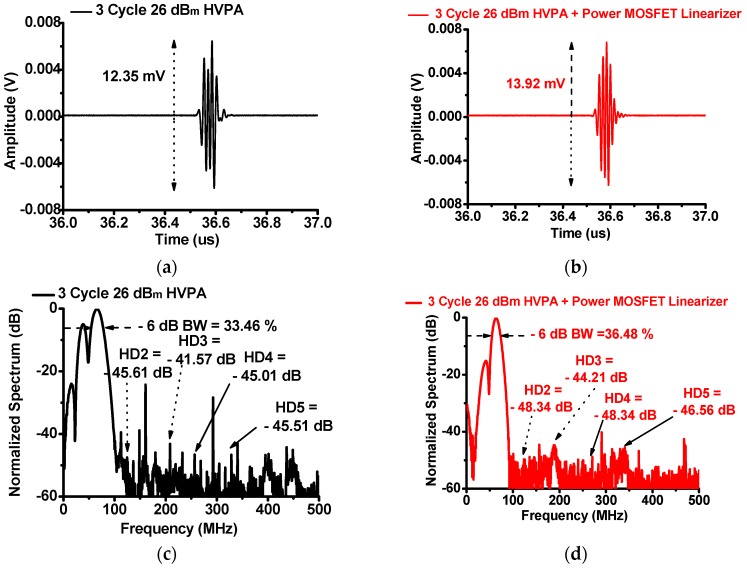
The echo amplitude of the HVPA (**a**) without and (**b**) with the power MOSFET linearizer and the spectrum of the HVPA (**c**) without and (**d**) with the power MOSFET linearizer when three-cycle 26 dB_m_ input power was applied.

Figure 10 shows the measured pulse-echo responses of the HVPA with and without the power MOSFET linearizer in the high-frequency pulse-echo instrumentation using a 75 MHz immersion-focused ultrasonic transducer (V3349, Olympus Inc., Shinjuku, Tokyo, Japan) when 75 MHz three-cycle 26 dB_m_ input power was applied to the function generated. In Figure 10a,b, the echo amplitude of the HVPA with the power MOSFET linearizer (13.92 mV) is higher than that of the HVPA without the power MOSFET linearizer (12.35 mV). In Figure 10c,d, the second, third, fourth, and fifth harmonic distortion components of a 75 MHz ultrasonic transducer (V3349) applied by the HVPA with the power MOSFET linearizer (−48.34, −44.21, −48.34, and −46.56 dB, respectively) are lower than those of the HVPA (−45.61, −41.57, −45.01, and −45.51 dB, respectively). The −6 dB bandwidth of the echo signal using the HVPA with the power MOSFET linearizer (36.48%) is increased compared to that using the HVPA without the power MOSFET linearizer (33.46%).

**Figure 11:**
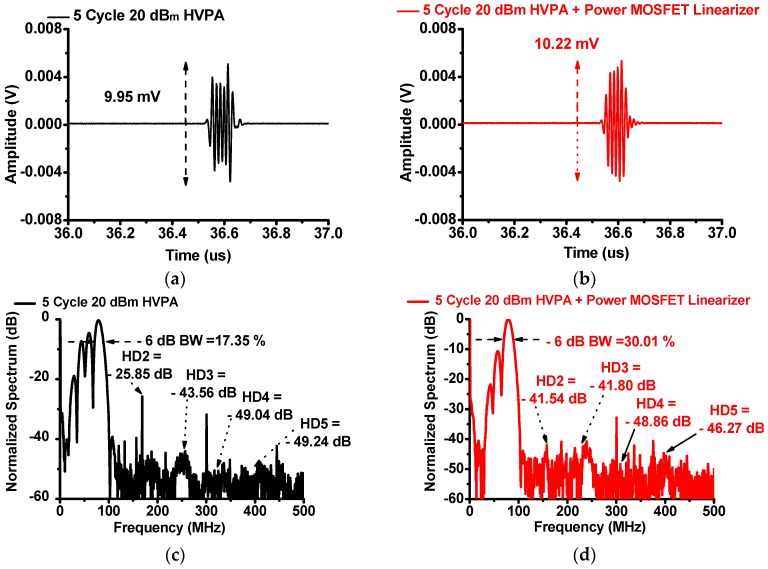
The echo signal amplitude when using the HVPA (**a**) without and (**b**) with the power MOSFET linearizer and the spectrum of the HVPA (**c**) without and (**d**) with the power MOSFET linearizer when five-cycle 20 dB_m_ input power is applied.

Figure 11 shows the measured pulse-echo responses of the HVPA with and without the power MOSFET linearizer using a 75 MHz immersion-focused ultrasonic transducer (V3349) when 75 MHz five-cycle 20 dB_m_ input power was applied. In Figure 11a,b, the echo amplitude of the HVPA with the power MOSFET linearizer (10.22 mV) is higher than that of the HVPA (9.95 mV). In Figure 11c,d, the second, third, fourth, and fifth harmonic distortions of the transducer applied by the HVPA with the power MOSFET linearizer (−41.54, −41.80, −48.86, and −46.27 dB, respectively) are lower than those of the HVPA without the power MOSFET linearizer (−25.85, −43.56, −49.04, and −49.24 dB, respectively). The −6 dB bandwidth of the echo signal using the HVPA with the power MOSFET linearizer (30.01%) increased compared to that using the HVPA without the power MOSFET linearizer (17.35%). As shown in the measurement results of the pulse-echo responses, the power MOSFET linearizer could reduce the unwanted harmonic distortion components and increase the amplitudes of the echo signal generated by 75 MHz ultrasonic transducers.

Finally, while circuit topologies and operation frequencies are different, making direct comparison difficult, Table 2 summarize the performances of our linearizer with that of selected previously reported linearizers.

## 4. Conclusions

Conventional HVPA used in the high-frequency pulse-echo instrumentation includes DC coupling capacitors to minimize the high-voltage DC effects on the high-frequency ultrasound transducer. However, this structure could reduce the gain of the HVPA with higher input powers. Harmonic distortions of the echo signals can severely limit the performance of high-frequency pulse-echo instrumentation. Therefore, a linear HVPA to trigger the ultrasonic transducer is highly desirable for such high-frequency pulse-echo instrumentation. Here, we proposed a novel power MOSFET linearizer in order to increase the linearity (reducing gain deviations) over a wide range of input power. The gain deviation of the power MOSFET linearizer has shown to have the opposite effect to that of the HVPA, thus increasing gain flatness over a wide input power range. Therefore, the power MOSFET linearizer can decrease the harmonic distortions of the HVPA, thereby reducing the unwanted harmonic distortions of the ultrasonic transducer in high-frequency pulse-echo instrumentation.

The structure of the power MOSFET linearizer is simple to implement because, in the power MOSFET linearizer structure, the MOSFET acts as a variable resistor to primarily affect the gain deviation performances. Therefore, the operation of the MOSFET under varying DC bias conditions in the power MOSFET linearizer was characterized for the HVPA. At 70 and 80 MHz, the measured gain deviation values of the HVPA with power MOSFET linearizer under 10 V DC bias voltage (−2.95 dB and −3.00 dB, respectively) were lower than that of the HVPA without power MOSFET linearizer (−1.80 dB and −1.11 dB, respectively). At 70 and 80 MHz, the measured IP_1dB_ point of the HVPA with power MOSFET linearizer under 10 V DC bias voltage (24.17 and 26.19 dB_m_, respectively) were higher than that of the HVPA without power MOSFET linearizer (22.03 and 22.13 dB_m_, respectively). In order to verify the capability of the power MOSFET linearizer, the echo signal harmonic distortions of the 75 MHz ultrasonic transducer were measured. Using 75 MHz 5-cycle 20 dB_m_ input power, the echo signal harmonic distortions using HVPA with power MOSFET linearizer (−41.54, −41.80, −48.86, and −46.27 dB, respectively) were measured to be lower than those of HVPA without the power MOSFET linearizer (−25.85, −43.56, −49.04, and −49.24 dB, respectively). We conclude that the power MOSFET linearizer has proven to be a simple, yet effective, technique for the HVPA to reduce unwanted harmonic distortions in the echo signals while providing higher echo signal amplitude and even wider echo signal bandwidth.

## Figures and Tables

**Table 1:** Summary of the performances of the HVPA with and without the power MOSFET linearizer under various DC bias voltages.

	HVPA without Power MOSFET Linearizer	HVPA with the Power MOSFET Linearizer (DC Bias Voltages)				
		1 V	3 V	6 V	9 V	10 V
Gain deviation at 70 MHz (dB)	−2.95	−2.25	−2.16	−2.01	−1.85	−1.80
Gain deviation at 80 MHz (dB)	−3.00	−1.37	−1.31	−1.11	−1.01	−0.96
IP_1dB_ at 70 MHz (dB_m_)	22.03	23.43	23.56	23.88	23.97	24.17
IP_1dB_ at 80 MHz (dB_m_)	22.13	24.43	24.85	25.89	26.16	26.19

**Table 2:** Performance comparison of our linearizer and a previously-reported design, including pulse-echo performances with ultrasound transducers.

Parameters	[13]	[14]	Our Design
Output power	55.1 dB_m_	41.59 dB_m_	37.6 dB_m_
Operating frequency	5 MHz	140 MHz	80 MHz
Second harmonic distortion (HD2)	−50 dB	−24.8 dB	−41. 54 dB
Third harmonic distortion (HD3)	-	-	−41.80 dB
